# Massive Atrial Thrombosis: A Serious Complication of the Maze Procedure Triggered by Heparin-Induced Thrombocytopenia (HIT)

**DOI:** 10.7759/cureus.41568

**Published:** 2023-07-08

**Authors:** Arwa Battah, Iyad Farouji, Abdelhadi Farouji, Reshma John, Preet Randhawa, Joaquim Correia

**Affiliations:** 1 Internal Medicine, Saint Michael's Medical Center, Newark, USA; 2 Internal Medicine, Assuta Ashdod Medical Center, Ben Gurion University of the Negev, Ashdod, ISR; 3 Internal Medicine, St George's University School of Medicine, West Indies, GRD; 4 Cardiology, Trinitas Hospital, Elizabeth, USA; 5 Cardiology, Saint Michael's Medical Center, Newark, USA; 6 Electrophysiology, Saint Michael's Medical Center, Newark, USA

**Keywords:** multimodality cardiac imaging, heparin induced thrombocytopenia, massive thrombosis, cox-maze, atrial fibrillation

## Abstract

Atrial fibrillation is one of the most common cardiac arrhythmias, classically presenting with an “irregularly irregular” rhythm with or without chest pain, palpitations, shortness of breath, lightheadedness, or fatigue. The maze procedure is an open-heart operation that creates a carefully designed maze of incisions and ablations in the atrial myocardium. Although it is a common procedure, serious complications may happen. Herein, we report on a 76-year-old man who presented with chest pain and atrial fibrillation and was found to have multi-vessel disease on a coronary angiogram. He underwent coronary artery bypass and the COX-maze procedure, which was complicated by a massive thrombosis in the atria and the superior vena cava following the ablation line, secondary to heparin-induced thrombocytopenia, which is extremely rare. The central focus of this paper is to present this rare complication to stress the importance of rigorous follow-up and anticoagulation therapy in patients undergoing the maze procedure. To our knowledge, we are the first to report such a rare case of diffuse large atrial thrombi triggered by heparin-induced thrombocytopenia (HIT) type II after a COX-maze procedure.

## Introduction

Atrial fibrillation is the most common arrhythmia, presenting in approximately 10% of patients over the age of 60 [1]. It can cause significant complications, including systemic thromboembolism and tachycardia-induced cardiomyopathy, leading to a diminished quality of life with increased morbidity and mortality [2]. Different treatment methods, from pharmacological to interventional, have been used to manage this disease. The maze procedure, a surgical procedure that is not guided by electrophysiological mapping, has been used as a treatment modality for atrial fibrillation for many years [3]. While beneficial in this aspect, it does come with its own set of complications. Herein, we present a 76-year-old gentleman who underwent a maze procedure that was complicated by heparin-induced thrombocytopenia (HIT), leading to diffuse thrombosis in the atria and the superior vena cava adherent to the surgical line.

## Case presentation

**Figure 1:**
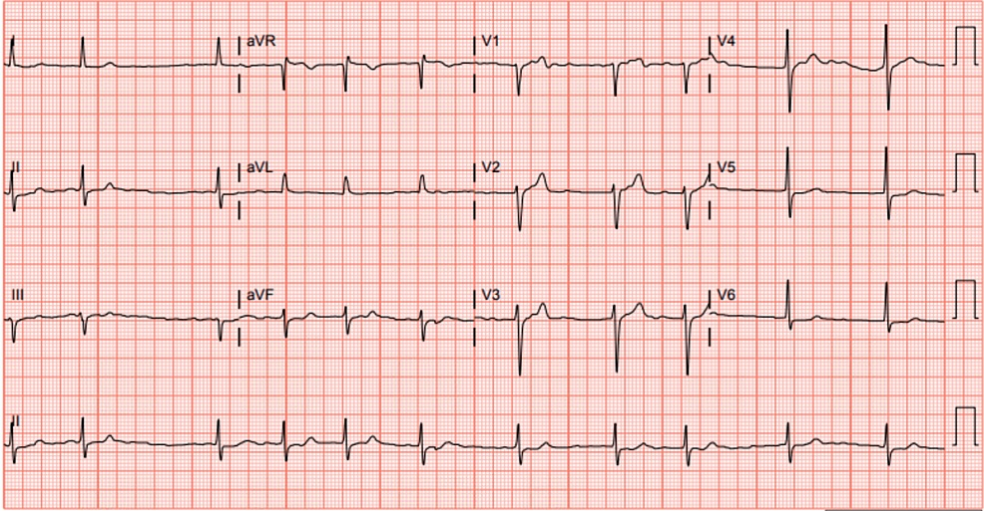
ECG showing atrial fibrillation.

A 76-year-old male presented to our emergency room for chest pain and progressively worsening shortness of breath for the last three weeks. He described the chest pain as a constant, left-sided, pressure-like pain, exacerbated by exertion and mildly relieved with rest. His medical history was significant for new-onset paroxysmal atrial fibrillation, hypertension, type II diabetes mellitus, and hyperlipidemia. A review of systems was unremarkable. He underwent a coronary angiogram at an outside hospital two weeks prior to his presentation to our facility, which revealed three-vessel coronary artery disease with significant lesions in the proximal right coronary artery (RCA), the proximal left anterior descending artery (LAD), with good relative distal targets, and the ramus intermedius. The cardiology team was consulted and recommended the patient undergo open heart surgery, which was not performed at that time. During the initial physical examination, he was hypertensive (143/59 mmHg) but otherwise comfortable. An initial EKG showed atrial fibrillation with borderline left axis deviation and no ST changes, with a rate of 71 beats per min (BPM). Aside from a low hemoglobin of 12.5 g/dL, (normal range 13.5-17.5 g/dL) and a mildly reduced glomerular filtration rate (GFR) of 85 mL/min (normal range: more than 90 mL/min), all other labs were unremarkable. A chest X-ray showed no acute cardiopulmonary disease. During the hospital stay, he underwent coronary artery bypass graft (CABG) surgery during which the left internal mammary artery (LIMA) was grafted to the LAD, and the saphenous vein was grafted to the ramus intermedius and distal RCA. He also underwent a complete left and right-sided radiofrequency maze procedure and left atrial appendage (LAA) ligation. The immediate postoperative period was unremarkable, and he was transferred to the cardiac recovery unit intubated and sedated. We started him on a therapeutic dose of heparin for anticoagulation. The postoperative ECG revealed a normal sinus rhythm. After the surgery, the patient was hypoxic, his oxygen saturation dropped to 89%, and he was unable to be extubated. A follow-up chest X-ray indicated new left-sided infiltrates, and he was started on antibiotics to treat pneumonia. Three days after the procedure, he was found to be in atrial fibrillation again (Figure 1) and was started on amiodarone, metoprolol succinate, and a heparin drip. A follow-up transthoracic echocardiogram showed an improved ejection fraction (EF) of 40-45% with normal right ventricular systolic function, a moderately dilated left atrium, and no significant valvular disease. Ten days after the procedure, his platelet count dropped from 240,000 per microliter on admission to 52,000 per microliter (normal range: 150,000-450,000 per microliter). This raised the suspicion of heparin-induced thrombocytopenia (HIT), with a 4T score of 7; therefore, the heparin drip was switched to an agratroban drip. The diagnosis of HIT was confirmed by a positive heparin-platelet factor 4 (PF4) by enzyme-linked immunoassay (ELISA) (41 ng/ml) and a positive platelet serotonin release assay (SRA).

**Figure 2:**
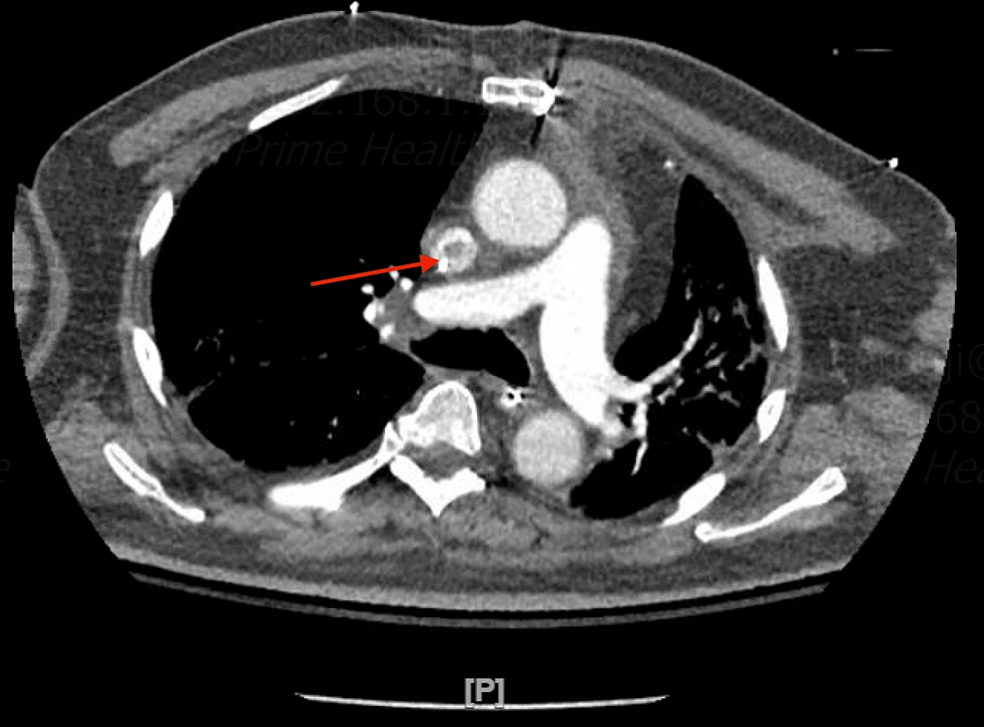
CTA of the chest revealed SVC thrombosis. CTA: computerized tomographic angiogram; SVC: superior vena cava

**Figure 3:**
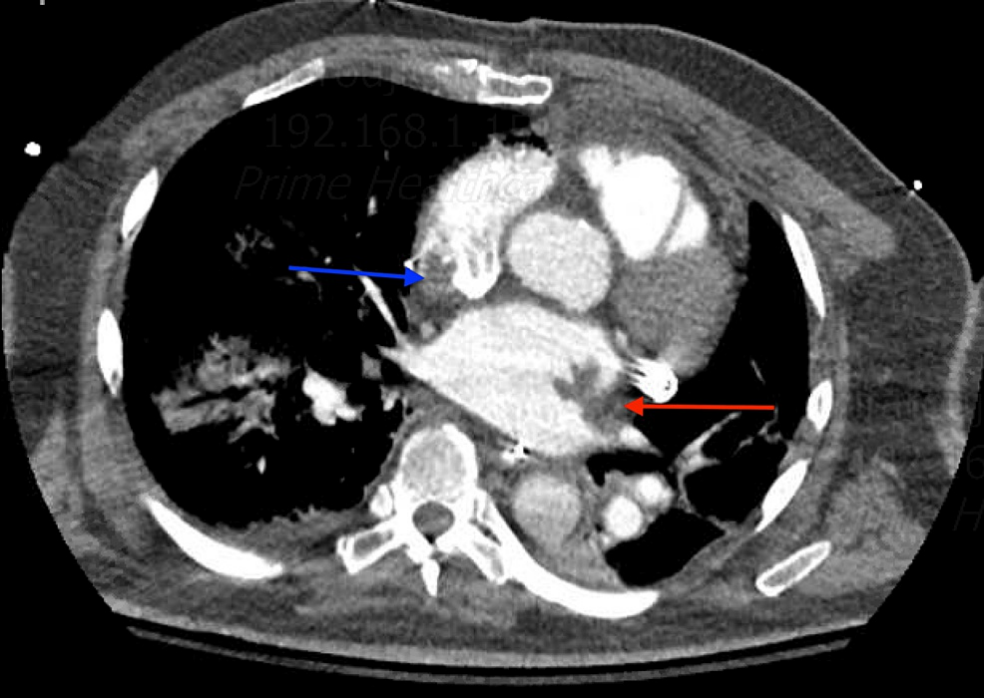
CTA of the chest showing extensive thrombosis in the left atrium (red arrow) and the right atrium (blue arrow) CTA: computerized tomographic angiogram

**Figure 4:**
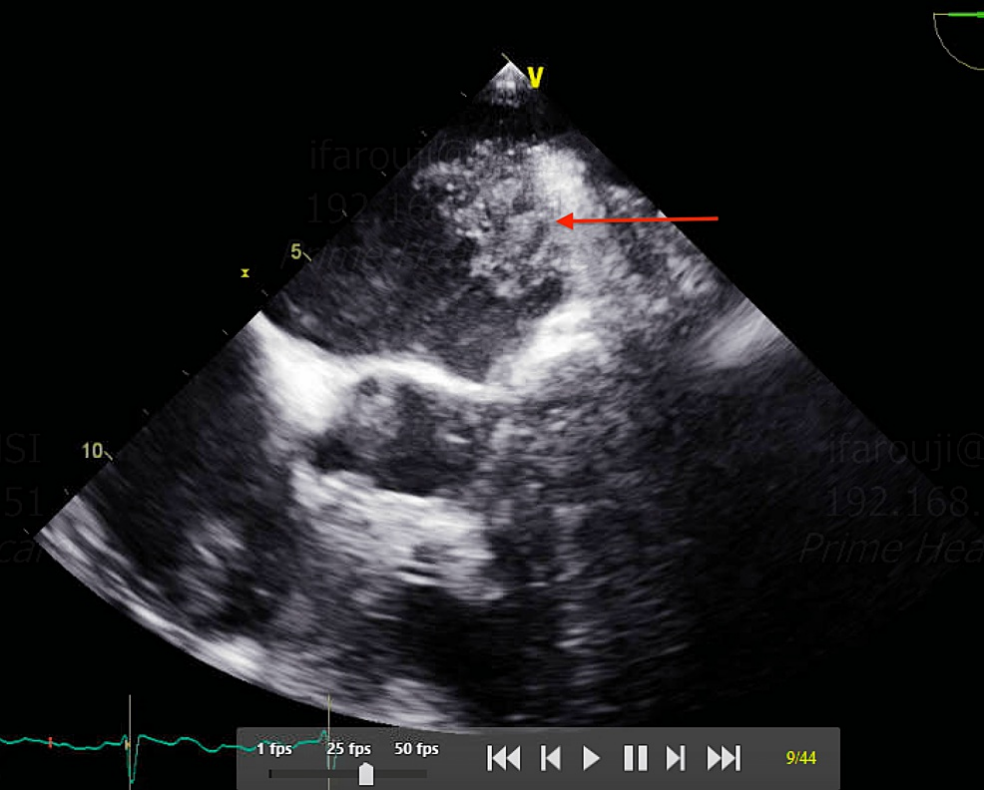
TEE showing a thrombus attached to the interatrial septum from the right side (red arrow) TEE: transesophageal echocardiogram

**Figure 5:**
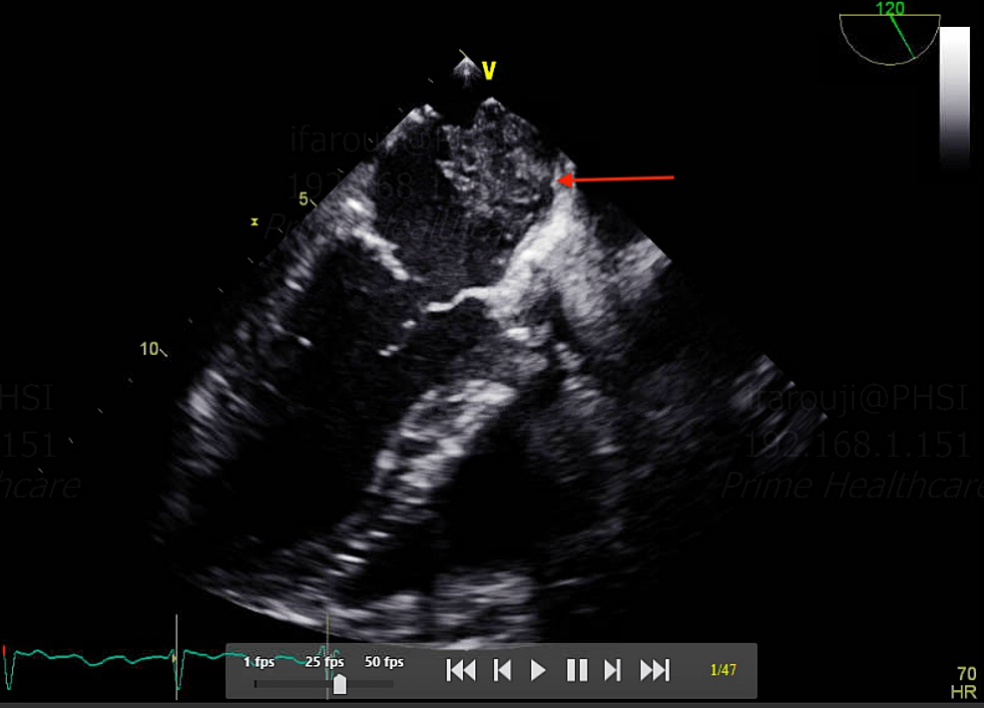
TEE showing a thrombus in the left atrium (red arrow) TEE: transesophageal echocardiogram

**Figure 6:**
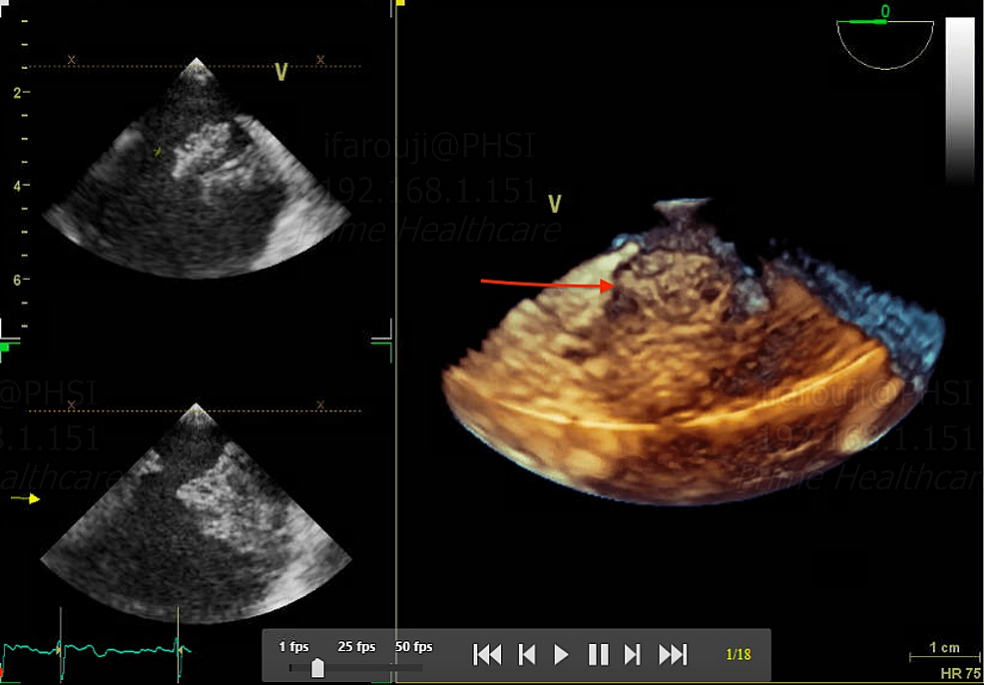
TEE, 3D reconstruction, showing a thrombus attached to the interatrial septum TEE: transesophageal echocardiogram

As the patient remained hypoxic and continued spiking fevers, he underwent a computerized tomographic angiogram (CTA) of the chest, which revealed small sub-segmental pulmonary emboli in the right lower lobe, with filling defects in the superior vena cava (SVC) and both atria (Figures 2, 3). A follow-up transesophageal echocardiogram (TEE) was performed for better assessment of the CTA findings, which showed multiple thrombi in both atria and the SVC following the pattern of the cryoablation that he underwent in the maze procedure (Figures 4-6). Later on, the patient was extubated successfully and was discharged to a rehabilitation center on Eliquis 5 mg twice daily, with a recommendation to follow up as an outpatient. His platelets went up to 131,000 on the day of the discharge.

## Discussion

Atrial fibrillation is the most common type of cardiac arrhythmia, and its prevalence continues to rise. Although the worldwide prevalence of atrial fibrillation is approximately 1%, the prevalence in those over the age of 75 is approximately 9% [4]. While atrial fibrillation may be a permanent disease, various treatments and risk-modifying strategies have been developed to treat and prevent the sequelae of this condition, many of which can lead to strokes. Such treatments include medications to control the coagulation, rate, rhythm, and interventions such as cardioversion, ablation, and other interventional cardiac procedures [1]. This report will focus on a serious complication of the maze procedure, one of the surgical approaches to atrial fibrillation management [1].

The maze procedure is a surgical treatment that divides the macro-reentry circuits in both atria, interrupting the circuit loop contributing to atrial fibrillation, and channels the impulses from the sinoatrial (SA) node to the atrioventricular (AV) node [5]. With synchrony reestablished, this procedure alleviates the three physiologic sequelae of AF (tachycardia, thromboembolism, and hemodynamic compromise) [2]. The restoration of sinus rhythm alleviates tachycardia and palpitations while the restoration of atrial contraction and AV synchrony leads to improved hemodynamics with a decreased risk of thromboembolism. Furthermore, antiarrhythmic and anticoagulant medications can be discontinued, though there are still no clear guidelines on when to stop these medications after the procedure [6]. Approximately 80-85% of patients who underwent the cryomaze procedure did not require antiarrhythmic therapy at 12 to 24 months postoperatively [7].

Even though the maze procedure cured AF, it was found that >40% of patients who underwent the procedure experienced early postoperative atrial tachyarrhythmias [3]. However, these atrial tachyarrhythmias were found to have different mechanisms than preoperative atrial fibrillation. One such mechanism is that post-procedure, there is an increase in the dispersion of atrial refractoriness, which can initiate electrical reentry into the atria [8]. Another mechanism is that the severe inflammatory response typically seen after surgery contributes to the occurrence of early postoperative ATA [9]. In our case, the patient developed pneumonia, which, in addition to his other risk factors, triggered postoperative atrial fibrillation.

Venous and arterial thrombi formation are well-known complications of HIT type II [10], but there are no clear reports of the percentage of patients who developed intracardiac thrombosis secondary to HIT. While approximately 25% to 50% of postoperative cardiac surgery patients develop antibodies to PF4-H (platelet factor 4 and heparin) complexes in their peripheral blood, only 1% to 3% develop clinical signs of HIT type II [11]. Cardiac procedures increase the risk of intracardiac thrombosis, as interruptions to the endocardium expose the underlying tissue and thereby increase the risk of initiation of the coagulation and thrombotic cascades, leading to thrombus formation [12]. Developing HIT type II, as in our case, after intracardiac surgery can be a significant trigger for the formation of thrombi. Herein, we are reporting a gentleman who developed HIT type II, which triggered the formation of massive and diffuse thrombi in the left atrium near the LAA, right atrium, and superior vena cava thrombi. The pattern of thrombus formation was found to follow the surgical path of the maze procedure and LAA ligation.

The ability of the maze procedure to dramatically decrease the risk of stroke associated with atrial fibrillation is likely due to the restoration of sinus rhythm and atrial transport function, in combination with surgical removal or obliteration of the left atrial appendage, where most thrombi associated with atrial fibrillation develop [13]. Despite that, there is still a significant percentage of surgical patients who experience embolic events postoperatively, which leads to hesitation by physicians to stop anticoagulation, especially in the absence of clear guidelines [14]. The literature encourages the use of different factors in assessing the need for anticoagulation, including the patient’s CHA2DS2-VASc score, rhythm status, echocardiographic findings, and risk of bleeding [15]. In our case, the patient developed postoperative atrial fibrillation that required the initiation of a heparin drip for anticoagulation measures. Unfortunately, he developed HIT type II and developed large and diffuse thrombosis in both atria and the superior vena cava. To our knowledge, we are the first to report a case of diffuse large atrial thrombi triggered by HIT type II after a maze procedure with LAA ligation, which makes our case a unique study.

## Conclusions

The maze procedure creates a carefully designed maze of incisions in the atrial myocardium that interferes with the electrical circuits of atrial fibrillation. The Cox-maze procedure has proved particularly useful for patients with preoperative atrial fibrillation, who are undergoing other types of other open-heart surgeries. While the procedure has been beneficial in the treatment of atrial fibrillation, serious complications can arise. In the case above, we discussed a patient who developed HIT after undergoing a maze procedure, leading to diffuse thrombosis in both the atria and superior vena cava. We are reporting this case to emphasize the importance of careful follow-up and anticoagulation therapy, as well as to address these rare postoperative thrombotic complications.
